# Data on the epitope mapping of soybean A2 and A3 glycinin

**DOI:** 10.1016/j.dib.2016.05.027

**Published:** 2016-05-21

**Authors:** Hanaa Saeed, Christine Gagnon, Elroy Cober, Steve Gleddie

**Affiliations:** Agriculture and Agri-Food Canada, Ottawa Research & Development Centre, Ottawa, Ontario K1A 0C6, Canada

**Keywords:** Epitope mapping, Soybean, Glycinin, Western blot

## Abstract

The data information provided in this article relate to our research article “Using patient serum to epitope map soybean glycinins reveals common epitopes shared with many legumes and tree nuts” (Saeed et al., 2016) [1]. Here we provide western blot detection of glycinin subunits by soy-sensitive human sera, ELISA screens with overlapping synthetic peptides (epitope mapping), and various database/server epitope searches.

**Specifications Table**

**Table:** 

Subject area	Immunology
More specific subject area	Allergy
Type of data	Tables, Graphs, Figures
How data was acquired	Western blots were performed by screening total soy protein on 2D gels with soy-sensitive human sera and detecting with a secondary anti-IgE-HRP antibody.
	ELISAs were performed by screening a collection of synthetic peptides encompassing the glycinin sequences with soy-sensitive human sera. The IgE binding to the peptides was detected by a secondary anti-IgE-HRP antibody.
	Epitope sequence similarity searches were done using the SDAP website: (http://fermi.utmb.edu/)
	B-cell epitope predictions were done using the following servers:
	ABCpred (http://www.imtech.res.in/raghava/abcpred/)
	BepiPred 1.0 (http://www.cbs.dtu.dk/services/BepiPred/)
	SVMTriP (http://sysbio.unl.edu/SVMTriP/)
Data format	Raw, analyzed
Experimental factors	Human serum samples were acquired from individuals that exhibited a sensitivity to soybean and to other legumes/nuts
Experimental features	Western blot, ELISA (epitope mapping)
Data source location	Canada and USA
Data accessibility	Data is provided with this article

**Value of the data**

• Better understanding of soy storage protein allergens may contribute to allergy management strategies.
• It may also contribute to the generation of hypoallergenic soybean cultivars.
• Provide risk assessment tools for the evaluation and characterization of the allergenicity of novel foods.

## 1 Data

**Fig. 1:**
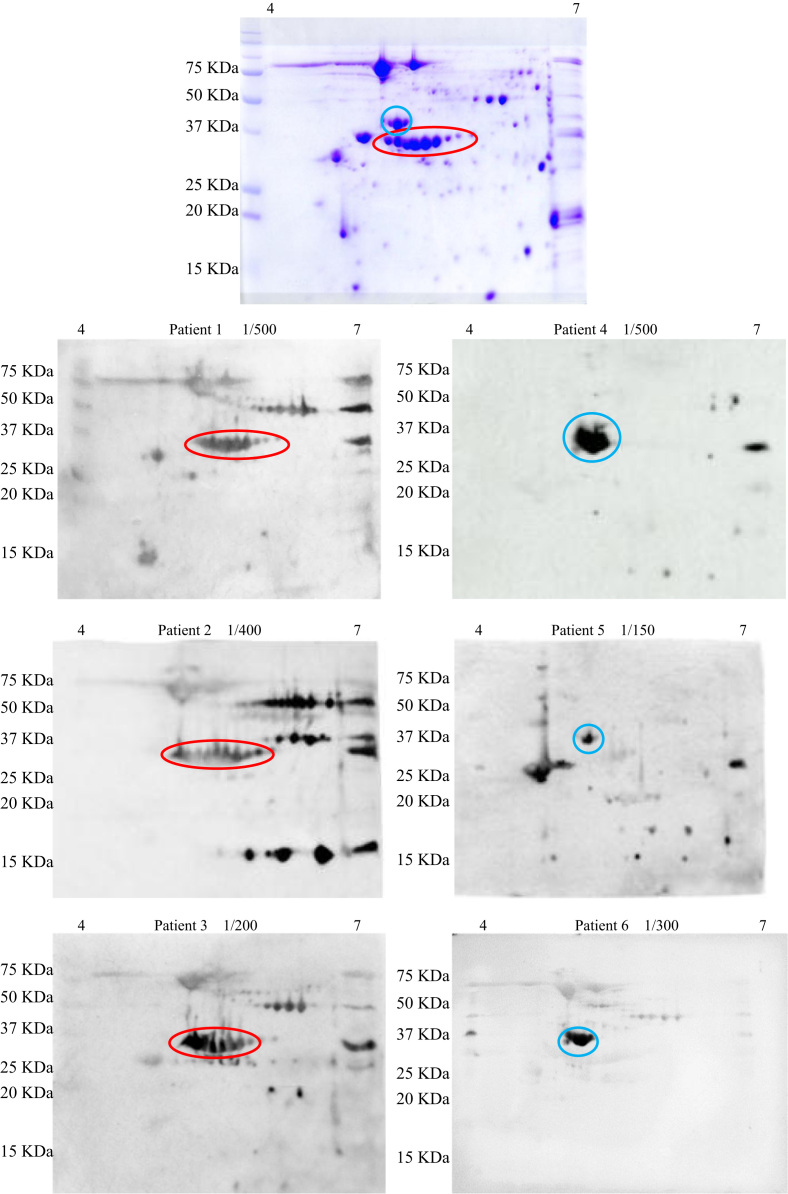
2D western blots of patient sera on soybean seed protein gels (bottom 6 panels). Top panel is a Coomassie-stained 2D gel illustrating the A1/A2 (red) and A3 (blue) glycinins.

**Fig. 2:**
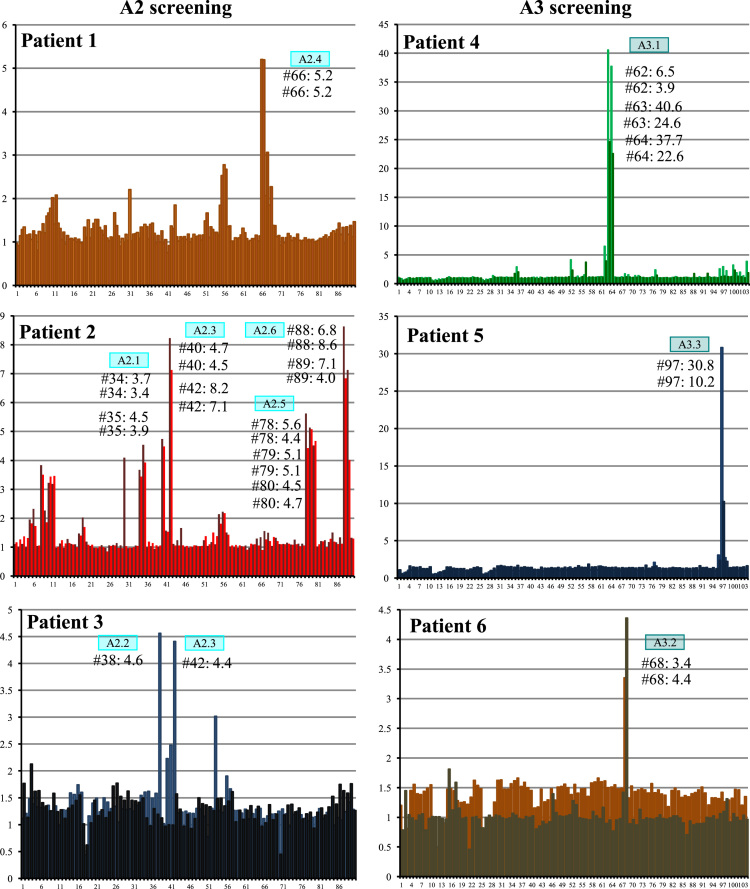
Epitope mapping of A2 and A3 Pepsets with patient serum 1–6. *X*-axis indicates Pepset peptide number and *y*-axis indicates ratio of colorimetric detection in the patient sample vs control sample.

**Fig. 3:**
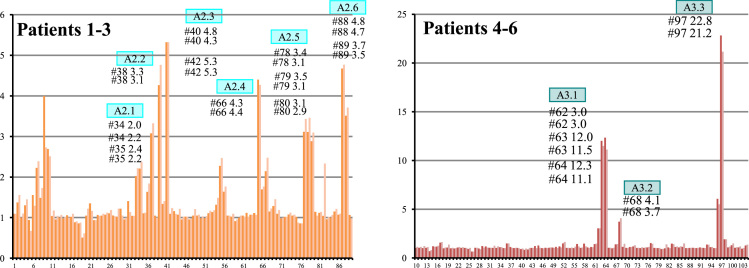
Epitope mapping of A2 and A3 Pepsets using pooled sera. *X*-axis indicates Pepset peptide number and *y*-axis indicates ratio of colorimetric detection in the patient sample vs. control sample.

**Fig. 4:**
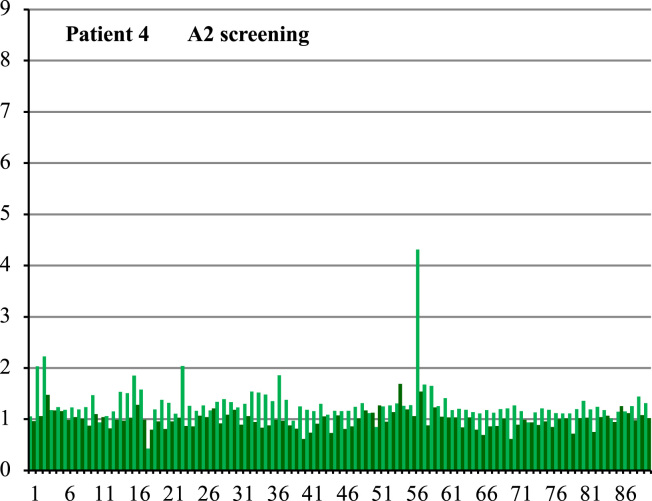
Serum specificity control. Screening of A2 Pepset with Patient 4 serum which only bound to A3 subunit on western blot (see Fig. 1). *X*-axis indicates Pepset peptide number and *y*-axis indicates ratio of colorimetric detection in the patient sample vs control sample.

The data presented here show the western blot detection of A2 or A3 subunits by soy-sensitive human sera (Fig. 1) and ELISA screens (Fig. 2, Fig. 3) of these patient sera with overlapping synthetic peptides (Pepsets). Serum specificity is also confirmed by cross-screening the A2 Pepset with a serum that does not bind to the A2 cluster on western blot (Fig. 4). Also contained in this article is SDAP (Structural Database of Allergenic Proteins) sequence similarity search results (Table 1, Table 2) of the epitopes reported by Saeed et al. (2016) [1] and theoretical B-cell epitope prediction data on the full length sequences of A2 and A3 subunits (Table 3).

## 2 Experimental design, materials and methods

### 2.1 Patient serum

Soy-sensitive human sera used in the western blots and epitope mapping are previously described [1].

### 2.2 Immunoblot analysis

Western blotting of human sera was conducted as previously described [2]. Membranes were hybridized with serum dilutions ranging from 1/50 to 1/500.

### 2.3 Epitope mapping

Two peptide sets representing the mature amino acid sequences of glycinin A2 (P04405, 90 peptides) and A3 (BAB15802, 104 peptides) were synthesized and biotinylated by Mimotopes (http://www.mimotopes.com) via parallel array platform. Quality Control Assurance was provided for both peptide synthesis and biotinylation by reverse phase HPLC (RP-HPLC), and by mass spectrometry (MS) respectively. The biotinylated 12-mer peptides, frame-shifted by three residues were used as per manufacturer׳s instructions (Application/Method PT3013). DMSO was used to resuspend the dry peptides and streptavidin-coated high capacity plates (Pierce #15500) pre-blocked with SuperBlock™ buffer were used to capture the biotinylated peptides. Serum was diluted at 1/50 in TBS-BSA 2% except for Patients 4 (1/100) and 5 (1/50 or 1/100). The secondary mouse anti-human IgE-HRP (Southern Biotech, Birmingham, Alabama, #9160-05) was diluted at 1/4000 in TBS-BSA 2%. SureBlue Reserve ^TM^ TMB microwell peroxidase substrate (KPL, Gaithersburg, Maryland, #53-00-01) was added to the plate, the reaction was stopped by acidification and colorimetric detection was performed on a Tecan Sunrise microplate reader with Magellan™ data analysis software (Tecan group AG, Männedorf, Switzerland) at 450 nm. Each experiment was performed in duplicate. Negative controls were performed using the same protocol, but the addition of human sera was omitted. The data was normalized by calculating the ratio of experimental to negative control and graphed.

### 2.4 B-cell epitope prediction servers

Three popular B-cell epitope prediction servers were tested with the A2 and A3 sequences. ABCpred server predicts B cell epitopes using a recurrent neural network (machine based technique) using fixed length patterns [3]. Lengths of epitopes varying from 10–16 amino acids were tested. BepiPred 1.0 server uses a combination of a hidden Markov model and a propensity scale method [4]. SVMTriP uses support vector machine integrating tri-peptide similarity and propensity scores [5], where epitope lengths varying from 10–20 amino acids were tested. In all cases, a higher score reflects a higher probability that a sequence is an epitope.

## Figures and Tables

**Table 1:** SDAP sequence similarity of A3 epitopes (food only).⁎

**Rank**	**Allergen**	**Source**	**PD index**	**Location**	**Matching region**
**1**	**A3.1 epitope**	**Soybean**	**0.00**	**214–222**	**KQGQHQQQE**
2	A4	Soybean	1.15	214–222	KQGQHQQEE
3	Vig r 2.0201	Mung bean	3.82	218–226	QQGQESQQE
4	Tri a glutenin	Wheat	3.83	191–199	QQGQSQQQQ
5	Pru du 6.01	Almond	3.87	100–108	QQGRQQEQE (epitope HS#5)
6	Pru du 6.01	Almond	3.87	136–144	QQGRQQQEE (epitope HS#5)
9	Pru du 6.02	Almond	3.87	209–217	QQGRQQQQQ (epitope)
10	A1a	Soybean	4.02	205–213	QKGKHQQEE
12	Pru du 6.01	Almond	4.06	122–130	QQGQQEQQQ (epitope HS#5)
14	Tri a gliadin	Wheat	4.14	23–31	QQQQQQQQE
15	Tri a gliadin	Wheat	4.34	142–150	KQQQQQQQQ
16	Tri a gliadin	Wheat	4.48	260–268	QQPQQQQQQ
18	Tri a gliadin	Wheat	4.84	208–216	HQQQQQQQE
20	Bra j 1	Indian mustard	4.93	88–96	QQGQQLQHE
21	Bra r 1	Field mustard	4.95	134–142	QQGQQQQMQ
22	Tri a gliadin	Wheat	4.97	119–127	QQAQQQQQQ
29	β-conglycinin (α′)	Soybean	5.16	433–441	EQQQRQQQE
**1**	**A3.2 epitope**	**Soybean**	**0.00**	**226–237**	**GSVLSGFSKHFL**
2	A4	Soybean	0.00	227–238	GSVLSGFSKHFL
3	Len c 1.0101	Lentil	3.80	144–155	PSFLSGFSKNIL
4	A1a	Soybean	4.26	218–229	GSILSGFTLEFL (epitope)
6	A1b	Soybean	4.66	217–228	GSILSGFAPEFL
7	Len c 1.0102	Lentil	4.71	144–155	PSFLSGFNKSIL
8	Ara h 3	Peanut	5.39	237–248	GNIFSGFTPEFL (epitope)
11	Vig r 2	Mung bean	5.86	183–194	QSYLQGFSKNIL
12	β-conglycinin (α′)	Soybean	5.86	366–377	QSYLQGFSKNIL
14	Pru du 6.01	Almond	6.16	269–280	NNVFSGFNTQLL
16	A2	Soybean	6.17	215–226	SNILSGFAPEFL (A2.5 epitope)
18	Vig r 2	Mung bean	6.30	181–192	QSYLRGFSKNIL
19	β-conglycinin (α)	Soybean	6.46	331–342	QSYLQGFSRNIL
21	Fag e 1	Buckwheat	6.49	241–252	ANILSGFQDEIL
22	Jug r 4	English walnut	6.54	231–242	NNVFSGFDADFL (epitope)
23	Cor a 9	Hazelnut	6.57	238–249	NNVFSGFDAEFL (epitope)
24	Car i 4	Pecan	6.57	232–243	NNVFSGFDAEFL (epitope)
25	Ara h 1	Peanut	6.57	304–315	SSYLQGFSRNTL
**1**	**A3.3 epitope**	**Soybean**	**0.00**	**313–324**	**EEEDQPRPDHPP**
2	A4	Soybean	4.84	316–327	EDEDKPRPSRPS
3	Lup an 1.0101	Lupin	7.00	101–112	EQEQQPRPQRRQ

⁎ Only scores up to 8 are indicated.

**Table 2:** SDAP sequence similarity of A2 epitopes (food only).⁎

**Rank**	**Allergen**	**Source**	**PD index**	**Location**	**Matching region**
**1**	**A2.1 epitope**	**soybean**	**0.00**	**121–129**	**QRPQDRHQK**
4	A1a	soybean	1.36	124–132	SRPQDRHQK
5	A1b	soybean	1.36	121–129	SRPQDRHQK
6	Tri a gliadin	wheat	4.72	241–249	QQPQQQQQQ
8	Pru du 6.02	almond	4.99	137–145	EDQQDRHQK
9	Pis v 5	pistachio	4.99	126–134	SRFQDKHQK
10	Ara h 3	peanut	5.01	138–146	QQQQDSHQK
11	Ana o 2	ashew	5.22	118–126	GRFQDRHQK (2aa-epitope)
12	Lup an 1	Lupine	5.27	126–134	QRPQSRREE
15	Cor a 9	Hazelnut	5.59	138–146	RSEQDRHQK (epitope)
16	Jug r 4	English walnut	5.70	210–218	RRQQQRQQR (epitope)
17	A4	Soybean	5.83	129–137	QQLQDSHQK
18	A3	Soybean	5.83	129–137	QQLQDSHQK
19	Ses i 7	Sesame	5.83	141–149	RRFMDRHQK
20	Tri a gliadin	Wheat	5.86	134–142	QQQQQQQQK
21	Lup an 1	Lupine	5.90	51–59	QQPRPRQQE
24	Car i 4	Pecan	6.14	131–139	EFQQDRHQK (epitope)
25	Jug r 4	English walnut	6.14	130–138	EFQQDRHQK (epitope)
26	Ber e 2	Brazil nut	6.18	128–136	GRFQDQHQK
27	Car i 4	Pecan	6.28	210–218	HRRQQQHQQ (epitope)
28	Lup an 1	Lupine	6.32	583–591	AQPQQQQQR
29	A1a	Soybean	6.45	115–123	QQPQQRGQS
31	β-conglycinin (α′)	Soybean	6.46	147–155	PRPHQPHQK
34	A2	Soybean	6.51	109–117	QEPQESQQR
35	Tri a gliadin	Wheat	6.56	210–218	QQQQQQEQK
37	Tri a glutenin	Wheat	6.58	191–199	QQGQSQQQQ
38	Vig r 2	mung bean	6.60	330–338	QREQQKQQE
39	Tri a gliadin	wheat	6.60	92–100	QQPQQQQQL
40	Ana o 1	cashew	6.61	42–50	QRQYDEQQK
42	Ara h 6	peanut	6.63	54–62	TRSSDQQQR
43	Tri a gliadin	wheat	6.65	137–145	QQQQQKQQQ
46	Jug r 2	English walnut	6.78	111–119	QRGRDRQDP
**1**	**A2.2 epitope**	**Soybean**	**0.00**	**130–141**	**VHRFREGDLIAV**
4	Ara h 3	Soybean	1.31	124–135	VHRFDEGDLIAV
6	A1b	Soybean	2.71	130–141	IYHFREGDLIAV
8	A1a	Soybean	2.77	133–144	IYNFREGDLIAV
9	Ses i 6	Sesame	3.21	144–155	VHRLRQGDIVAI
10	Pis v 5	Pistachio	3.40	135–146	IQRFRKGDIIAL
11	Ana o 2	Cashew	4.02	127–138	IRRFRRGDIIAI
12	Cor a 9	Hazelnut	4.08	147–158	IRHFREGDIIAL (5aa-epitope)
13	Pis v2	Pistachio	4.25	151–162	VRHIREGDIIAL
14	Car i 4	Pecan	4.31	140–151	IRHFREGDIIAF
15	Jug r 4	English walnut	4.31	139–150	IRHFREGDIIAF (5aa-epitope)
16	Ber e 2	Brazil nut	4.58	137–148	VHHLKKGDIIAI
17	Pru du 6.02	Almond	4.70	146–157	IRHIREGDIIAL
18	Pru du 6.01	Almond	4.70	193–204	TRRIREGDVVAI
20	Ses i 7	Sesame	4.73	150–161	VRQFRQGDILAL
21	Pis v2	Pistachio	5.84	146–157	VRPIQEGDVIAL
22	Sin a 2	White mustard	6.61	181–192	VEHVRHGDAIAM
23	Fag e 1	Buckwheat	6.94	161–172	IFRIREGDVIPS
24	A3	Soybean	7.07	138–149	IRHFNEGDVLVI
25	A4	Soybean	7.07	138–149	IRHFNEGDVLVI
**1**	**A2.3 epitope**	**Soybean**	**0.00**	**136–153**	**GDLIAVPTGVAWWMYNNE**
2	A1a	Soybean	0.00	139–156	GDLIAVPTGVAWWMYNNE
5	A1b	Soybean	1.22	136–153	GDLIAVPTGFAYWMYNNE
6	Ara h 3	Peanut	2.21	133–150	GDLIAVPTGVAFWLYNDH
9	Pis v2	Pistachio	4.67	157–174	GDIIALPAGVAHWIYNNG
11	Ber e 2	Brazil nut	4.92	143–160	GDIIAIPAGVALWCYNDG
12	Pru du 6.02	Almond	4.92	152–169	GDIIALPAGVAYWSYNNG
13	Cor a 9	Hazelnut	5.10	153–170	GDIIALPAGVAHWCYNDG
14	Car i 4	Pecan	5.26	146–163	GDIIAFPAGVAHWCYNDG
15	Ana o 2	Cashew	5.40	133–150	GDIIAIPAGVAHWCYNEG
16	Pru du 6.01	Almond	5.43	179–196	GDVVAIPAGVAYWSYNDG
18	Ses i 6	Sesame	5.75	150–167	GDIVAIPSGAAHWCYNDG
19	Jug r 4	English walnut	5.80	145–162	GDIIAFPAGVAHWSYNDG
20	Pis v 5	Pistachio	6.10	141–158	GDIIALPAGVANWCYNEG
21	Ses i 7	Sesame	6.18	156–173	GDILALPAGLTLWFYNNG
22	A3	Soybean	7.83	144–161	GDVLVIPPGVPYWTYNTG
23	A4	Soybean	7.83	144–161	GDVLVIPPGVPYWTYNTG
**1**	**A2.4 epitope**	**Soybean**	**0.00**	**214–225**	**GSNILSGFAPEF**
3	A1b	Soybean	1.52	216–227	GGSILSGFAPEF
4	Ara h 3	Peanut	2.76	236–247	GGNIFSGFTPEF (epitope HS#2)
7	Fag e 1	Buckwheat	4.61	240–251	GANILSGFQDEI
8	A1a	Soybean	4.81	217–228	GGSILSGFTLEF (epitope HS#2)
10	Cor a 9	Hazelnut	5.31	237–248	GNNVFSGFDAEF (epitope HS#2)
11	Car i 4	Pecan	5.31	231–242	GNNVFSGFDAEF (5aa-epitope HS#2)
12	Pru du 6.02	Almond	5.92	225–236	GNNIFSGFDTQL (epitope HS#2)
13	Sin a 2	White mustard	6.16	248–259	QQNILSGFDPQV
14	Jug r 4	English walnut	6.17	230–241	GNNVFSGFDADF (epitope HS#2)
15	A4	Soybean	6.17	226–237	GGSVLSGFSKHF
16	A3	Soybean	6.17	225–236	GGSVLSGFSKHF (A3.2 epitope)
17	Pru du 6.01	Almond	6.38	288–299	GNNVFSGFNTQL (epitope HS#2)
19	Ana o 2	Cashew	6.51	196–207	GRNLFSGFDTEL
20	Gly m Bd28K	Soybean	6.54	167–178	SHSVLSGFEPAI
22	Pis v2	Pistachio	7.21	229–240	SNNILSAFDEEI
25	Ses i 7	Sesame	7.67	225–236	TKNIFNGFDDEI
26	Gal d vitellogenin (Gal d 6)	egg	7.85	771–782	ANQILNSIAGQW
**1**	**A2.5 epitope**	**Soybean**	**0.00**	**256–261**	**KGGLRV**
3	Ara h 3	Peanut	2.11	279–284	RGGLRI (epitope HS#3)
7	A1a	Soybean	2.93	259–264	KGGLSV (epitope HS#3)
8	A1b	Soybean	2.93	258–263	KGGLSV
9	Pis v 5	Pistachio	4.47	253–258	KGDLQV
10	Sin a 2	White mustard	4.57	82–87	KGGLYL
11	Ana o 1	Cashew	4.83	394–399	KGGMSV
13	A3	Soybean	4.86	265–270	EGGLSV
14	A4	Soybean	4.86	266–271	EGGLSV
22	Cap a 1	Bell pepper	5.35	128–133	PGSLRV
33	Gal d vitellogenin (Gal d 6)	egg	5.67	1395–1400	TGGLQL
39	Gal d 6	Egg	5.87	49–54	RTGIRI
41	Lyc e 4	Tomato	5.90	167–172	ESGLHV
45	Api g 2	Celery	5.93	99–104	KCGIRI
46	Rub i 1	Red raspberry	5.96	114–119	KGGAEI
**1**	**A2.6 epitope**	**Soybean**	**0.00**	**283–291**	**QCVETDKGC**

⁎ Only scores up to 8 are indicated.

**Table 3:** B-cell epitope prediction.

Name	Amino acid	Sequence	ABCpred	Bepipred	SVMTriP
A3.1	214–222	KQGQHQQQE	0.72 (Rank 8)	1.63	–
A3.2	226–237	GSVLSGFSKHFL	–	–	0.737
A3.3	313–324	EEEDQPRPDHPP	0.81 (Rank 7)	2.72	–
A2.1	121–129	QRPQDRHQK	–	1.33	0.293
A2.2	130–141	VHRFREGDLIAV	0.90 (Rank 4)	–	0.428
A2.3	136–153	GDLIAVPTGVAWWMYNNE	0.87 (Rank 7)	–	0.319
A2.4	214–225	GSNILSGFAPEF	0.82 (Rank 7)	–	0.384
A2.5	256–261	KGGLRV	0.80 (Rank 9)	–	0.302
A2.6	283–291	QCVETDKGC	–	1.03	0.299

Amino acid number corresponds to position of full length sequence. A dash (–) indicates that no epitope was found. Scores are listed for the 3 different methods tested. Only the highest score is listed if the epitope was found in multiple lengths tested (10–20 mer).
