# Supplementary material for: Data on the epitope mapping of soybean A2 and A3 glycinin
Source: Data Brief. 2016 May 21;8:123–31. doi: 10.1016/j.dib.2016.05.027 (PMC4889876; doi:10.1016/j.dib.2016.05.027)
Supplement: Supplementary file 1 — Supplementary material [file mmc1.docx]

Conflict of Interest

The authors report no conflict of interest.
